# ‘*Candidatus* Liberibacter solanacearum’ distribution and diversity in Scotland and the characterisation of novel haplotypes from *Craspedolepta* spp. (Psylloidea: Aphalaridae)

**DOI:** 10.1038/s41598-020-73382-9

**Published:** 2020-10-06

**Authors:** Jason C. Sumner-Kalkun, Fiona Highet, Yvonne M. Arnsdorf, Emma Back, Mairi Carnegie, Siobhán Madden, Silvia Carboni, William Billaud, Zoë Lawrence, David Kenyon

**Affiliations:** 1 SASA, Roddinglaw Road, Edinburgh, EH12 9FJ Midlothian UK; 2 University of Glasgow, Glasgow, G12 8QQ UK; 3 Dipartimento Di Agraria, Universita Degli Studi Di Sassari, Viale, Italia 39, 07100 Sassari, Italy; 4 INRAE, GAFL, 84140 Montfavet, France

**Keywords:** Molecular biology, Plant sciences, Ecology, Biodiversity, Microbial ecology, Microbiology, Bacteria, Microbial communities, Environmental microbiology, Microbial genetics, Zoology, Entomology

## Abstract

The phloem limited bacterium *‘Candidatus* Liberibacter solanacearum’ (Lso) is associated with disease in Solanaceous and Apiaceous crops. This bacterium has previously been found in the UK in *Trioza anthrisci*, but its impact on UK crops is unknown. Psyllid and Lso diversity and distribution among fields across the major carrot growing areas of Scotland were assessed using real-time PCR and DNA barcoding techniques. Four Lso haplotypes were found: C, U, and two novel haplotypes. Lso haplotype C was also found in a small percentage of asymptomatic carrot plants (9.34%, n = 139) from a field in Milnathort where known vectors of this haplotype were not found. This is the first report of Lso in cultivated carrot growing in the UK and raises concern for the carrot and potato growing industry regarding the potential spread of new and existing Lso haplotypes into crops. *Trioza anthrisci* was found present only in sites in Elgin, Moray with 100% of individuals harbouring Lso haplotype C. Lso haplotype U was found at all sites infecting *Trioza urticae* and at some sites infecting *Urtica dioica* with 77.55% and 24.37% average infection, respectively. The two novel haplotypes were found in *Craspedolepta nebulosa* and *Craspedolepta subpunctata* and named Cras1 and Cras2. This is the first report of Lso in psyllids from the Aphalaridae. These new haplotypes were most closely related to Lso haplotype H recently found in carrot and parsnip. Lso was also detected in several weed plants surrounding carrot and parsnip fields. These included two Apiaceous species *Aegropodium podagraria* (hap undetermined) and *Anthriscus sylvestris* (hap C)*;* one *Galium* sp. (Rubiaceae) (hap undetermined); and *Chenopodium album* (Amaranthaceae) (hap undetermined).

## Introduction

‘*Candidatus* Liberibacter solanacearum’ (Lso) is an emerging pathogen associated with disease in economically important crop plants within the Apiaceae and Solanaceae. Lso is vectored by insects in the family Psylloidea, also called “psyllids” or “jumping plant-lice”. Lso is divided into genetic groups based on unique SNPs in ribosomal RNA gene regions. These are known as “haplotypes” which have different geographic ranges and plant hosts; being closely linked to the range and feeding behaviour of the psyllid vector. Ten haplotypes of Lso have previously been described in the literature (A^1^, B^1^, C^2^, D^3^, E^4^, F^5^, G^6^, H^7^, H(Con)^8^, and U^9^), with four (C, D, E and H) associated with disease in a wide range of Apiaceous crops such as carrot^7,10^, parsley^11^, parsnip^9,11^, celery^4^, fennel and chervil^11^. Haplotype C is found in Northern Europe mainly vectored by *Trioza apicalis* and the combination of pathogen infection and psyllid feeding can cause up to 100% loss of Apiaceous crops in Finland^12,13^ and Sweden^14^. The distribution of this vector species has not been extensively studied in the UK. From publicly available records^15^ and a small number of specimens found in suction trap catches^16^, *T. apicalis* appears to be more common in Southern England and has not been reported in Scotland.

Lso haplotypes D and E are vectored mainly by *Bactericera trigonica*^17–19^; both haplotypes and their psyllid vectors can be found in the Mediterranean region^4,20^, North-Western Europe^11,21^, Israel^19^ and Northern Africa^22–25^, but these haplotypes or their vectors are not known to be present in the UK^15^. Haplotype E has been found in infected commercial parsley seed sold in the UK^26^, but these seed lots did not originate from the UK. Two haplotype Hs have been described due to concurrent publication, these two H haplotypes are genetically distinct and have different psyllid and plant hosts. The first haplotype H was described from symptomatic carrot in Finland and was also found in parsnip and native weed plants in the Polygonaceae (*Fallopia convolvulus* and *Persicaria lapathifolia*)^7^. The psyllid vector for this haplotype is currently unknown. The second haplotype H, referred to henceforth as H(Con), was first found infecting plants in the Convolvulaceae, including *Convolvulus arvensis* (field bindweed) and *Ipomea batatas* (sweet potato)^27^. H(Con) haplotype was assigned after further analysis of 16S ribosomal gene regions^8^ but the full set of ribosomal genes necessary for assignment of new haplotypes has not been sequenced.

Haplotypes, A, B and F are associated with disease in Solanaceous plants, with A and B mainly associated with disease in potato and tomato and vectored by the psyllid *Bactericera cockerelli* in North America^1^, Central America^10,28–30^, New Zealand^31^, and South America^32^*.* This psyllid is currently an A1 quarantine pest in the EPPO region and absent from the UK^33^. Haplotype G was found in 49 year-old U.S. herbarium specimens of *Solanum umbelliferum*^6^. Haplotype U was first found in symptomatic *Urtica dioica* (stinging nettle) in Finland and is vectored by the psyllid *Trioza urticae* but is not yet known to cause economic damage^9^.

Most psyllid species are confined to a very narrow host range, only able to utilize one host plant or closely related plant species^34^. However, Lso transmission to new plant hosts may arise when a psyllid species can use multiple host or food plants or is introduced to a new area, such as is the case for *B. cockerelli*^35–37^ and *B. trigonica*^17^*.* Concerns have arisen over the movement of psyllids onto non-host plants where feeding may occur incidentally or when their host plant is not available. Although *B. cockerelli* and *B. trigonica* are not considered effective vectors on crops other than their host plants^18,38^, these psyllids are capable of low-level Lso transmission to non-host crops such as carrot for *B. cockerelli*^38^, and potato and tomato for *B. trigonica*^17,18^. Although no experimental evidence has been found that *T. apicalis* can transmit haplotype C to potato plants^39^, haplotype C has been found within the stolons of volunteer potatoes and *Solanum nigrum* in Finland^9,40^. Lso has also been found in *Bactericera nigricornis and Bactericera tremblayi* in Spain^18^; and an unidentified *Bactericera* sp. from Tenerife^41^. The role of these psyllids in Lso transmission is not fully understood but one study suggests that *B. nigricornis* and *B. tremblayi* are not effective vectors^42^.

Currently only Lso haplotype C has been found to be present in the UK from Lso positive *Trioza anthrisci* specimens caught in suction trap samples in Elgin, Moray, and the small number of *T. apicalis* that were caught in a suction trap survey of the UK were negative for Lso^43^. MLST analysis has shown significant differences between haplotype C found in *T. apicalis* and carrot hosts compared to haplotype C found in *T. anthrisci* and *Anthriscus sylvestris* hosts^9^. The ability for *T. anthrisci* to transmit Lso and cause symptoms in carrot is currently unknown. To assess the impact of Lso haplotype C found in *T. anthrisci* and the extent of Lso infection in Scotland*,* surveys were performed across the major carrot growing regions in Scotland to determine the distribution of *T. anthrisci* and other psyllids and plants associated with Lso. The focus of this study was carrot and parsnip as these crops are associated with Lso haplotype C damage in Finland, Sweden and Norway^7,14,44,45^, albeit within a different psyllid vector. Solanaceous crops were not surveyed as associated haplotypes have not been found in the UK in psyllids or plants. Potential wild plant reservoirs were also screened for the presence of psyllids and Lso to assess the risk of transmission from weed to crop by psyllids or other vectors.

## Results

### Psyllid diversity in carrot fields and field margins

**Figure. 1:**
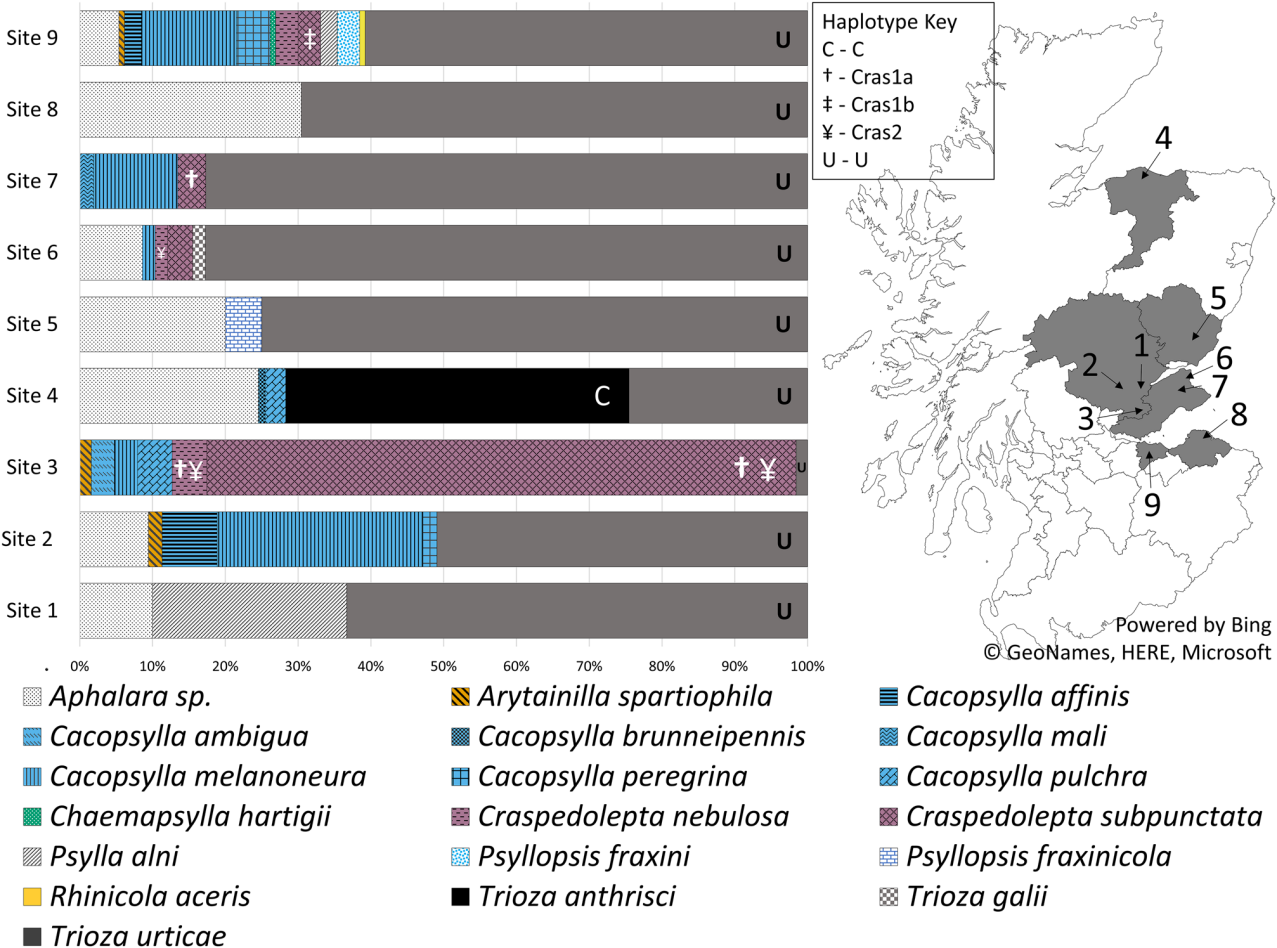
Psyllid diversity and psyllid associated Lso haplotypes found at nine different locations sampled in this study. The most abundant and most commonly identified psyllid was *T. urticae* (Supp Table 1), which was collected at all locations and was infected with Lso haplotype U (40.91%-100% infection rates). *Trioza anthrisci* was found only from locations in Elgin and was infected with haplotype C (100% infection rate). At three locations *C. subpunctata* and *C. nebulosa* were found to be infected with novel Lso haplotypes (Cras1a, Cras1b and Cras2). Cras1a and Cras1b are genetic variants of the same haplotype Cras1. Shetland, Orkney and the Outer Hebrides were not sampled, and therefore are not represented on the map. Counties where at least one sampling event took place are greyed. Locations: 1 = Scone; 2 = West Perth; 3 = Milnathort; 4 = Elgin; 5 = Forfar; 6 = Tayport; 7 = Howe of Fife; 8 = Tyninghame; 9 = SASA Farm. Map was created using Microsoft Excel, Microsoft product screen shot reprinted with permission from Microsoft Corporation.

In order to understand the diversity of plant and insect hosts associated with Lso and the risk of spill-over to crops, plants and psyllids in field margins were surveyed along with psyllids and plants from carrot or parsnip crops across Scotland’s major carrot growing areas. Sites surveyed are as follows: 1 = Scone, Perth and Kinross; 2 = West Perth, Perth and Kinross; 3 = Milnathort, Perth and Kinross; 4 = Elgin, Moray; 5 = Forfar, Angus; 6 = Tayport, Fife; 7 = Howe of Fife, Fife; 8 = Tyninghame, East Lothian; and 9 = SASA Farm, Midlothian. A total of 535 specimens comprising 19 psyllid species were found in carrot fields and the surrounding vegetation (Fig. 1. & Supp Table 1). In all but two locations (sites 3 and 4), *T. urticae* was the most abundant psyllid species found in field margins. At site 3, *Craspedolepta subpunctata* was the most abundant psyllid in field margins and was mainly collected from Rosebay Willow Herb (*Chamaenerion angustifolium*). At site 4, *T. anthrisci* was the most abundant psyllid species; found on carrot plants and *Anthriscus sylvestris*. *Trioza anthrisci* were only collected from site 4 which is close to the suction trap location where Lso infected *T. anthrisci* were previously collected in Elgin^43^. Eight other species not known to feed on carrot were found on carrot crops but in low numbers (Table 1).

**Table 1:** Total numbers of psyllids collected directly from carrot plants via sweep net across the field season (April 2018–Sep 2018) at each location. In 7 out of the 9 sites psyllids were present on carrot plants.

Location	Psyllid species	# on carrot
Scone, Perth and Kinross (site 1)	*Aphalara* sp.	3
	*Psylla alni*	8
	*Trioza urticae*	3
West Perth, Perth and Kinross (site 2)	*Aphalara* sp.	5
	*Trioza urticae*	1
Milnathort, Perth and Kinross (site 3)	*Cacopsylla ambigua*	2
	*Cacopsylla pulchra*	2
	*Trioza urticae*	1
Elgin, Moray (site 4)	*Aphalara* sp*.*	26
	*Cacopsylla ambigua*	1
	*Cacopsylla brunneipennis*	1
	*Cacopsylla pulchra*	3
	*Trioza anthrisci*	50
	*Trioza urticae*	1
Forfar, Angus (site 5)	None collected	0
Tayport, Fife (site 6)	*Aphalara* sp.	5
	*Trioza galii*	1
	*Trioza urticae*	2
Howe of Fife Valley, Fife (site 7)	*Cacopsylla mali*	1
	*Trioza urticae*	1
Tyninghame, East Lothian (site 8)	None collected	0
SASA Farm, Midlothian (site 9)	*Aphalara* sp*.*	2

The highest numbers of psyllids found on carrot were *T. anthrisci* and *Aphalara* sp*.* at Moray location. Other species were found in low numbers and were possibly incidental on the crops.

### Lso distribution and diversity in psyllid hosts and characterization of new haplotypes

All psyllids collected were tested for Lso using real-time PCR and Lso haplotypes were identified by PCR and in-house Sanger sequencing. The proportion of psyllids testing positive for Lso varied by location and species (Table 2). All *T. anthrisci* tested were positive for Lso haplotype C (n = 13, average C_t_ = 33.14 ± 4.28 s.d.) (Table 2). *Trioza urticae* was found associated with Lso haplotype U in all sites surveyed. However, the proportion of *T. urticae* found to harbour Lso U varied between site ranging from 41 to 100% infection.

**Table. 2:** ‘*Candidatus* Liberibacter solanacearum’ (Lso) distribution and infection levels associated with psyllids collected from carrot/ parsnip fields and surrounding vegetation in Scotland.

Psyllid Species	Location	Number tested	% Lso positives	Lso haplotype (% of positives)	Gene Region Accession #			
					16S	16-23S	50S	OMP
*Craspedolepta nebulosa*	Milnathort (site 3)	3	100	Cras1a (33.33)	MT229445	MT230487	MT249166	MT238961
				Cras2 (66.66)	MT229461	MT230506	MT249167	MT238975
	Tayport (site 6)	1	100	Cras2 (100)	MT229462	MT230507	MT249172	MT238976
	SASA Farm (site 9)	4	0	N/A	N/A	N/A	N/A	N/A
*Craspedolepta subpunctata*	Milnathort (site 3)	40	55.81	Cras1a (86.96)	MT229447-48, MT229452-60	MT230488-90, MT230492-93, MT230498-504	MT249168-69, MT249188-89, MT249202-05, MT249207-12	MT238962-64, MT238966-67, MT238970-74
				Cras2 (13.04)	MT229463	MT230508-09	MT249190, MT249206	MT238977-78
	Howe of Fife (site 7)	2	100	Cras1a (100)	MT229449	MT230494-95	MT249175-76	/
	SASA Farm (site 9)	4	75.00	Cras1b (100)	MT229446, MT229450-51	MT230491, MT230496-97, MT239505	MT249171, MT249177-78, MT249217	MT238965, MT238968-69
*Trioza anthrisci*	Elgin (site 4)	13	100	C (100)	MT229433-44	MT230482-86	MT249194-201, MTMT249214-16, MT249220	MT238956-60
*Trioza urticae*	Scone (site 1)	19	73.68	U (100)	MT229471	/	/	/
	West Perth, (site 2)	27	40.91	U (100)	/	/	MT249174	/
	Milnathort (site 3)	1	100	U (100)	/	/	/	/
	Elgin (site 4)	26	83.33	U (100)	/	/	/	/
	Forfar (site 5)	15	78.95	U (100)	MT229472	MT230522	/	/
	Tayport (site 6)	48	74.47	U (100)	/	/	MT249173	/
	Howe of Fife (site 7)	43	97.87	U (100)	MT229473-74	MT230514, MT230523-24	MT249170, MT249191- 92	/
	Tyninghame (site 8)	16	100	U (100)	/	/	MT249213	/
	SASA Farm (site 9)	79	48.75	U (100)	/	/	MT249174	/

Infection levels differed between psyllid species and between locations. *Trioza urticae* was infected with Lso in all sites but with different infection levels. New haplotypes Cras1 and Cras2 were found from three of the sites in *C. nebulosa* and *C. subpunctata*. Haplotype C was only found in *T. anthrisci* from Elgin, Moray. “/” = no sequence obtained.

Two novel Lso haplotypes were found in two psyllid species not previously known to harbour Lso. The SNPs for new haplotypes were characterised in 4 different gene regions including 16S rRNA (OA2/ Lsc2^12,46^); 16–23s IGS (Lp Frag 4-1611F/ Lp Frag 4-480R^47^); 50s *rplJ/rplL* (CL514F/ CL514R^48^) ; and outer membrane protein (OMB1482F/ OMB2086R^5,49^) (Supp Tables 2–5). Compared to its closest known relative (hap H), Cras1 showed 1 SNP difference in the 16S rRNA gene region; 3 SNPs difference in the 16–23S region; and 6 SNPs in the 50S region. Compared to its closest known relative (hap H), Cras2 showed 1 SNP difference in the 16s rRNA gene region; 1 SNP difference in the 16–23s region; and 2 SNPs in the 50S region. These SNPs justify them being considered new haplotypes. The two novel haplotypes were found in *C. nebulosa* and *C. subpunctata* at sites 3, 8 and 9 (Fig. 1 and Table 2). These are the first findings of Lso in Aphalaridae as hitherto all Lso haplotypes have been found in psyllids of the Triozidae family. We propose the haplotype names Cras1 and Cras2 named after the psyllid genus in which they were first found; further reasoning for this will be covered in the discussion.

A further variant of Cras1 was also found in specimens of *C. subpunctata* which differs to other Cras1 sequences by 3 SNPs in the 50 s rplJ/rplL gene region (Supp Table 4) but is identical to Cras1 in all other gene regions sequenced in this study (Supp Tables 2, 3 and 5). We distinguish between these two genetic types by naming them Cras1a and Cras1b. Haplotype Cras1a was found in *C. subpunctata* and *C. nebulosa* at site 3 and in *C. subpunctata* at site 7 (Fig. 1 and Table 2). Cras1b was found in *Cras. subpunctata* collected at site 9; whereas *C. nebulosa* collected from this location were negative for Lso. Cras2 was found in *C. subpunctata* and *C. nebulosa* at site 3; and in *C. nebulosa* at site 6 (Fig. 1 and Table 2). At site 3 where Cras1a and Cras2 haplotypes were present in both *Craspedolepta* spp., Cras2 was more common in *C. nebulosa* than Cras1a with 1:2 ratio (Cras1a:Cras2) of infected psyllids (Table 2). However, at site 3, Cras1a was more common in *C. subpunctata* than Cras2 with a 10:1 ratio (Cras1a:Cras2) (Table 2).

**Figure. 2:**
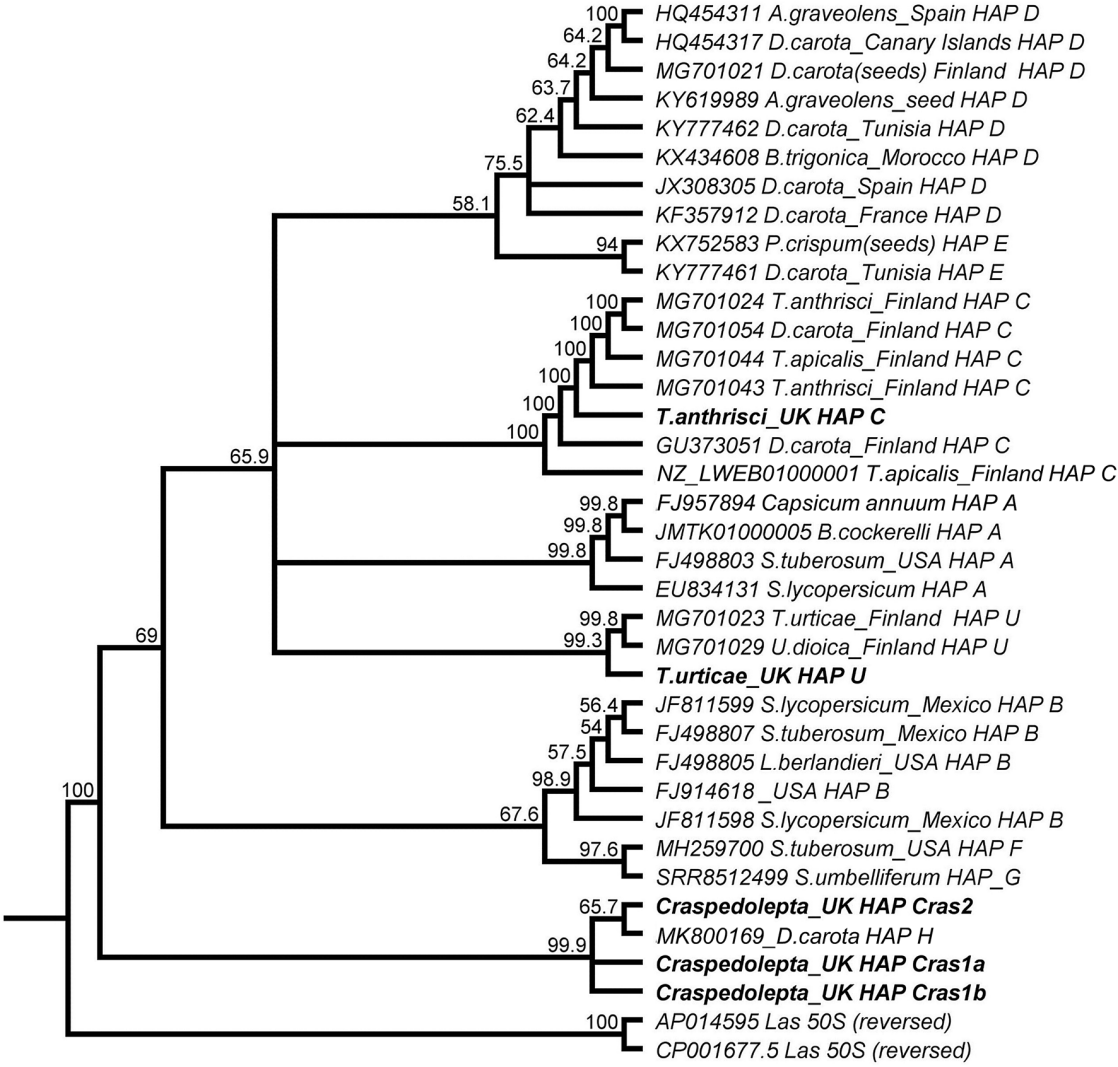
Maximum likelihood (50%) consensus phylogenetic tree of 50s *rpl*J/*rpl*L rRNA gene region (680 bp) of ‘*Candidatus* Liberibacter solanacearum’ (Lso) haplotypes, including those found in Scotland in the current study (bold). ‘*Candidatus* Liberibacter asiaticus’ (Las) was used as an outgroup. Tamura-Nei method was used for calculating distances and clustering was performed using UPGMA with 1000 bootstraps. New haplotypes Cras1 and Cras2 formed a separate clade with the recently described haplotype H from carrot in Finland. Numbers on branches represent the percentage of iterations that support that clade (1,000 replicates).

In phylogenetic analysis of the 50S rplJ/rplL gene region Cras1 variants and Cras2 formed a clade with the recently described haplotype H found in carrot in Finland^7^ (Fig. 2). All other described haplotypes (A, B, C, D, E, F, G and U) formed a separate clade. Accession numbers for all successfully sequenced gene regions were deposited into GenBank and can be found in Table 2.

### Lso distribution in plant hosts

A total of 587 plant samples from field margins were screened for Lso across nine survey sites. The plant families represented most commonly in field margins were Poaceae (Grasses) 39%, Apiaceae 13%, Onagraceae (represented by willowherb, *C. angustifolium*) 12%, and Urticaceae 11% (represented mainly by stinging nettle *U. dioica*). Lso positive plant material was found at six of the sites from six different plant species (Table 3).

**Table. 3:** A summary of results for Lso testing in carrots, parsnips and weeds from the nine field sites examined in this study.

Location	Plant Species	Common Name	# tested	Positives (haplotype)	% infection
Scone (site 1)	*Anthriscus sylvestris*	Cow Parsley	11	2	18.18
	*Daucus carota*	Wild Carrot	4	0	0.00
	*Daucus carota* subsp. *sativus*	Carrot	42	**0**	**0.00**
	*Urtica dioica*	Stinging Nettle	5	**1(U)**	20.00
West Perth (site 2)	*Daucus carota* subsp. *sativus*	Carrot	29	**0**	**0.00**
	*Urtica dioica*	Stinging Nettle	8	**3(U)**	37.50
Milnathort (site 3)	*Chamaenerion angustifolium*	Rose Bay Willowherb	35	**0**	**0**
	*Daucus carota* subsp. *sativus*	Carrot	139	**19(C)**	**9.34**
Elgin (site 4)	*Aegopodium podagraria*	Ground Elder	5	4	80.00
	*Anthriscus sylvestris*	Cow Parsley	4	**3(C)**	75.00
	*Chenopodium album*	Pigweed	2	2	100.00
	*Daucus carota* subsp. *sativus*	Carrot	74	**0**	**0.00**
	*Urtica dioica*	Stinging Nettle	5	1	20.00
Forfar (site 5)	*Daucus carota* subsp. *sativus*	Carrot	16	**0**	**0.00**
Tayport (site 6)	*Daucus carota*	Wild Carrot	6	0	0.00
	*Daucus carota* subsp. *sativus*	Carrot	14	**0**	**0.00**
Howe of Fife (site 7)	*Aegropodium podagraria*	Ground Elder	7	1	14.29
	*Anthriscus sylvestris*	Cow Parsley	20	3	15.00
	*Daucus carota*	Wild Carrot	2	0	0.00
	*Daucus carota* subsp. *sativus*	Carrot	15	**0**	**0.00**
	*Galium sp.*	Galium	4	2	50.00
	*Urtica dioica* subsp. *galeopsifolia*	Stingless Nettle	5	1	20.00
	*Urtica dioica*	Stinging Nettle	18	4	22.22
Tyninghame (site 8)	*Pastinaca sativa*	Parsnip	10	**0**	**0.00**
SASA Farm (site 9)	*Anthriscus sylvestris*	Cow Parsley	27	1	3.70

A small percentage of carrot plants were positive for Lso from Milnathort. Cultivated crop plants (carrot or parsnip) at all other sites were negative for Lso. The most commonly found plants to be infected were *U. dioica* (stinging nettle) and *A. sylvestris* (cow parsley); found at four sites each. Wild carrot plants were also tested from 3 sites but did not result in any positive samples.

Carrot (eight sites) and parsnip (one site) plants were sampled and positive carrot plants were found at site 3 with 9.34% infection levels (Table 3); average C_t_ was 37.74 suggesting low titres of Lso. Cultivated carrot plants at all other sites were negative for Lso. Lso was not detected in any parsnip plants tested. The haplotype detected in carrot was determined as hap C.

Lso infected weed plants were mainly from the Apiaceae (*Aegopodium podagraria, A. sylvestris*); but also included one *Galium* sp. (Rubiaceae); stinging nettle (*U. dioica*) and stingless nettle (*U. dioica* subsp. *galeopsifolia*); and *Chenopodium album* (Amaranthaceae) (Table 3). Stinging nettle (*U. dioica*) and cow parsley (*A. sylvestris*) were the most widespread plants infected with Lso, harbouring hap U and hap C respectively; each with positive plants found at four locations (Table 3).Two samples of *C. album* were tested and both were positive for Lso, however the sample size is too small to comment on infection rates in this plant and more samples will be tested. Infection rates of stinging nettle plants were relatively consistent across sites ranging from 20.00 to 37.50%.

Haplotyping of Lso was successful for only a subset of these positive weed plant samples (Table 3) due to low titres within plant material leading to difficulties with PCR amplification and sequencing of gene regions used for characterisation. Haplotypes found in cow parsley from site 4 were confirmed to be haplotype C. Lso found in stinging nettle from sites 1 and 2 were confirmed to be haplotype U. Despite finding a high number of Lso positive *C*. *nebulosa* and *C. subpunctata* feeding on willow herb (*C. angustifolium*) at site 3 we were unable to amplify Lso DNA from 35 plant samples tested (Table 3).

## Discussion

Our understanding of the diversity, distribution and host range of Lso continues to expand rapidly; in most cases in response to symptomatic crops and associated losses. Although symptomatic crops have not been reported in the UK, the recent finding of Lso haplotype C in *T. anthrisci* from suction trap samples collected in Scotland^43^ instigated this further study which aimed to better understand the risk posed to agriculture by examining the distribution of *T. anthrisci*, other psyllid vectors of Lso and potential natural plant reservoirs around agricultural sites. Our survey results give an indication of the natural distribution and diversity of Lso and associated plant/insect hosts in the absence of a noticeable impact on agriculture. However, our results pose further questions, such as: what is the source of infection of carrots at site 3 (Milnathort) as no known vector of haplotype C was detected at this site?

This study details the first finding of Lso in cultivated carrot crops in the U.K., along with first findings of Lso in Amaranthaceae, Rubiaceae and two psyllid hosts in the Aphalaridae. The finding of haplotype C within cultivated plants is of concern to carrot growers. However, titres of the bacterium were low, and plants did not exhibit symptoms at the time of collection in early September. It is unclear how Lso was transmitted to these plants as no known vectors were collected around this site and it is possible that another unknown vector may be responsible. Although it is not possible to rule out seed transmission, previous studies suggest that this is not a major pathway of Lso infection^50–52^.

*Trioza anthrisci* was the only psyllid found to harbour a haplotype known to pose risk to crops (haplotype C) in this survey in Scotland. This psyllid was found on carrot and *A. sylvestris* from site 4. This supports the previous finding of Lso infected *T. anthrisci* in suction trap samples from Elgin^43^. Negative results for the presence this species across all other sites indicate that the *T. anthrisci* population in Scotland is localised, although the finding of Lso haplotype C in carrot plants at a site approx. 130 miles away (site 3) suggests further UK-wide surveys of Lso and its vectors are advisable. Furthermore, previous studies suggest significant genetic differences between Lso haplotype C found in *T. anthrisci* and the devastating haplotype C found in *T. apicalis* and carrot^9^.

Known vectors of Lso on crops (such as *B. trigonica* or *T. apicalis*) were not detected at any site during this study. These species have not previously been recorded in Scotland^15,16^ and are therefore assumed to be absent from Scotland. The most northerly confirmed British record of *T. apicalis* comes from York, England^53^ which concurs with a finding of *T. apicalis* from suction trap samples from York, England^16^. Hodkinson and White^54^ suggest that *T. apicalis* is uncommon in the UK and is more abundant in Southern England. Given their patchy distribution and lack of evidence to suggest they cause economic damage in the UK, *T. anthrisci* and *T. apicalis* do not yet have an impact on agriculture in the UK. However, bearing in mind the recency of outbreaks in crops in areas of Europe^2,4,14,21,23,55^ and Africa^22^; continued monitoring of population changes and new introductions is recommended.

Haplotype U was first described from stinging nettle in Finland in 2018^9^. Within the current study haplotype U was found to be distributed widely across Scotland and associated with *T. urticae* and *U. dioica*, providing first confirmation that this haplotype is present in the UK and supporting previous speculation that it is present in other parts of Europe^56^. The wide distribution and 20–30% plant infection levels at all sites suggests that this is naturally occurring in Scotland and not a recent introduction. Low numbers of *T. urticae* were found on carrot in this study but infection of carrot with haplotype U was not observed, suggesting that these individuals were found on plants incidentally or, if carrot can be used as a casual food plant; *T. urticae* might be unable to transmit Lso U to carrot.

The current consecutive alphabetical naming convention for Lso haplotypes (except for haplotype U which was named after its host plant *Urtica dioica)* appears to be unfit for the rapid expansion in understanding of the bacterium. Firstly, it gives no indication to the host range or location; secondly this has led to confusion with two new haplotypes designated with the same name on more than one occasion. Two genetically different haplotypes were designated as haplotype “H” and published within 24hrs of each other^7,8^. One haplotype was found in carrot, parsnip, and Polygonaceae weed species in Finland^7^ and the other was found in *B. cockerelli* and Convolvulaceae^8^. Similarly, two manuscripts have named two different haplotypes G one being a peer reviewed paper describing Lso in *S. umbelliferum* in the USA^6^ and the other a non-peer reviewed article deposited in biorxiv.org describing Lso from carrot in France^57^. The former study has more credibility as it has been through the peer review process, the latter requires further characterisation in the ribosomal RNA genes to define its haplotype. To avoid further confusion and to distinguish the haplotypes described in this study we have named them Cras1 and Cras2 after the genus of psyllids in which they were first found: *Craspedolepta.* This should prevent the issue of studies that are being reviewed concurrently with this one that may have used the subsequent lettering system.

Hitherto psyllids known to be associated with Lso belonged to the Triozidae family. Psyllids harbouring novel haplotypes Cras1 and Cras2, *C. nebulosa* and *C. subpunctata*, belong to the Aphalaridae. This suggests further haplotypes of Lso may await discovery in other understudied psyllid species. Specimens of both psyllid species found at sites 3 and 6 were infected with either haplotype Cras1 or Cras2, co-infection with both haplotypes in the same psyllid specimen was not found in this study. However, it did appear that Cras1 was more commonly found in *C. subpunctata* and Cras2 was more commonly associated with *Cras. nebulosa*; the reason for this bias requires further study. Two distinct strains of Cras1 were identified in *C. subpunctata*, with differences in their 50S ribosomal gene sequences. These strains were from different locations (sites 3 and 9), approximately 26 miles apart, suggesting rapid evolution and isolated genetic communities of Lso may exist even over a relatively small area. The two sites are also separated by a large body of water which could act as a barrier to genetic flow between the two distinct populations.

Weed species and carrot plants from sites where Lso positive *Craspedolepta* spp. were found were tested for Lso, but neither Cras1 nor Cras2 were found in plants. The main host plant of *C. subpunctata* and *C. nebulosa* is *C. angustifolium*^34,54,58,59^ on which these psyllids have a broad north-circumpolar range and their populations regularly overlap^58^. Given the only known host plant of *C. nebulosa* and *C. subpunctata* is *C. angustifolium* it is likely that this plant is also infected with Cras1 and Cras2 haplotypes. However, we were unable to confirm this association in this study despite testing many samples of *C*. *angustifolium* (n = 35). The early immature stages of *C. nebulosa* and *C. subpunctata* overwinter on the roots of their host plant and emerge from the soil as later immature stages to develop into adults when ambient temperatures increase, and early shoots begin to develop^60^. It might be necessary to sample plant material earlier in the season to detect Lso in these plants or to find Lso within root material as thus far we have only sampled aerial parts of *C. angustifolium*.

The insect/ psyllid vector of haplotype H described from carrot in Finland is still to be determined but it is posited that it could also be a psyllid from the Aphalaridae such as *Aphalara borealis, A. freji* or *A. maculipennis*; inferred from their known plant host (*P. lapathifolia*)^7^. In our study we also observed that *Aphalara* spp. were commonly present on carrot plants. It is possible that haplotypes Cras1 and Cras2 could also infect carrot, given their genetic similarity to the recently described haplotype H which was found in carrot. However, during the current study, carrots at sites where Lso Cras1 or Cras2 positive psyllids were found (sites 3, 6 and 9) did not show infection with these haplotypes. Additionally, *C. nebulosa* and *C. subpunctata* were absent from carrot plants in all locations despite field margins often being less than 2 m away from cultivated crops and containing large amounts of these psyllids during the carrot growing season.

Recent studies have revealed that Lso is more genetically diverse than previously known, with a total of seven new haplotypes (including Cras1 and Cras2) being described within the last 3 years^5–9^; representing a more than twofold increase in the number of haplotypes known to science. It is almost certain that further haplotypes of Lso exist and are yet to be discovered; further work is required to ascertain their impact on agriculture, horticulture and the natural environment. Assignment of the correct haplotype in a transmission study is necessary to understand the differences in plant-Lso interactions, psyllid-plant hosts and agricultural risk of different Lso haplotypes. This is evident for a few new findings: including a possible new haplotype which was suggested by Contreras-Rendon et al.^8^ by reanalysing existing Lso sequences from a previous study in which *B. cockerelli* was found to transmit Lso to *C. arvensis* and *I. batatas*^27^. Transmission of Lso by *B. cockerelli* to these plants has not been demonstrated for Lso haplotypes A and B. Additionally, an unidentified *Bactericera* sp. caught on carrot in Tenerife was found to be positive for Lso but the haplotype was not characterised^41^.

The presence of Lso in weed species as found in this study is a potential source of Lso transmission to surrounding crop plants, although this phenomenon is generally not well understood. In this study we report the first instance of Lso in the Amaranthaceae and Rubiaceae. Lso was detected in the weed *C. album*, an ubiquitous weed species and known wild reservoir of a number of plant viruses^61^, suggesting Amaranthaceae could be a significant reservoir of Lso. The family Amaranthaceae includes many economically important crops such as spinach, chard, beetroot, sugar beet and quinoa. Further Lso testing should be performed on Amaranthaceae and the psyllid species associated with these plants.

Lso is present in psyllids, weeds and crop plant hosts in Scotland and is more genetically diverse than previously understood. Comparisons of different Lso haplotypes could lead to insights regarding gene expression and pathogenesis in their plant hosts. Understanding the distribution and diversity of Lso haplotypes and the psyllid vectors present in a region or country could help inform management and risk assessment of this bacterium. The risk and impact of Lso transmission in the UK appears to be low due to fragmentary populations of Lso vectors and low numbers of important psyllid species, such as *T. apicalis,* which are associated with Lso transmission and damage to crops. Continued monitoring should be undertaken to ensure invasions of non-native Lso vectors, such as *B. cockerelli* and *B. trigonica*, have not occurred and are dealt with quickly if they have. The difference between *T. apicalis* populations in mainland Europe and the UK are unknown, but introduction of *T. apicalis* from countries such as Austria, Finland, France, Germany, Netherlands and Norway via plant trade poses a significant threat to carrot production in the UK. Continued monitoring of carrot plants should be performed to ensure levels of Lso infection do not increase. Further study is needed to understand the risk of transmission of new haplotypes to important crop plants. Finally, a new convention for naming of haplotypes and minimum requirements for characterizing new haplotypes should be set-out. This study highlights the need to understand: 1) How many haplotypes of Lso have an impact on plant health? and 2) What are the fundamental differences between plant pathogenic haplotypes and those that are found as part of the natural microbiome of psyllids and/ or plants?

## Materials and methods

### Collection of samples

Nine field sites in Scotland were surveyed between April-December 2018 for Lso positive psyllids and Lso positive plants targeting psyllids found on Apiaceous crops surrounding carrot/parsnip fields and on the carrot crops themselves. Most fields were visited at least twice throughout the season. Psyllids were collected at 50 m intervals on a transect of the field margins, larger patches of Apiaceous weeds were also targeted if not otherwise included in the transect. Psyllids were collected in sweep-nets and were removed from the net in the field using a pooter and collected in 25 ml universal tubes. Psyllid specimens were frozen at − 20 °C before subsequent DNA extraction. Plant samples were taken from symptomatic and asymptomatic cultivated carrot or parsnip plants and any weed hosts that harboured psyllids. A sample of stem, lower leaf and upper leaf were taken from each plant sampled and stored at − 80 °C before DNA extraction. Sites surveyed are as follows: 1 = Scone, Perth and Kinross; 2 = West Perth, Perth and Kinross; 3 = Milnathort, Perth and Kinross; 4 = Elgin, Moray; 5 = Forfar, Angus; 6 = Tayport, Fife; 7 = Howe of Fife, Fife; 8 = Tyninghame, East Lothian; and 9 = SASA Farm, Midlothian (Fig. 1).

### DNA extraction: Psyllids

DNA was extracted from psyllids using a non-destructive method first described in^62^ adapted from^63^. Psyllid specimens were preserved in 95% Ethanol: 5% Glycerol solution. Using a 15 mm long, 0.15 mm diameter stainless steel entomological head-less pin (A3 size, Watkins and Doncaster) mounted in a holder, specimens were pierced fully through the abdomen and half-way through the thorax from the dorsal side while attempting to minimise damage to head, legs, wings, terminalia and other body parts that are used for taxonomic identification. Pierced specimens were then placed in a microcentrifuge tube containing 180 µl of ATL buffer and 20 µl of proteinase-k as outlined in the “DNeasy Blood and Tissue Kit from Animal Tissues” (Qiagen). Samples were placed in a shaking incubator over-night (~ 8–10 h) at 56 °C at 300 rpm. The subsequent steps of the above-mentioned protocol were then followed, and the psyllid integument voucher specimens were stored in 95% Ethanol: 5% Glycerol solution to allow subsequent morphological identification if necessary.

### DNA extraction: Plant

For plant DNA extractions, 100 mg samples were cut from the stem, upper leaf and lower leaf of each plant sample collected. Plant material was flash frozen and homogenised by shaking on a “TissueLyser II” (Qiagen) with 2 × 3 mm tungsten carbide beads added to each tube. DNA was extracted and purified following manufacturer guidelines for the “Biosprint 15 DNA Plant Kit” (Qiagen) and stored at − 80 °C until use in real-time PCR screening for Lso.

### Lso detection and characterisation

DNA extracted from Psyllids and plant material was screened for the presence of Lso using the real-time PCR assay described in Li et al.^64^. Lso DNA from positive samples was amplified using “Type-IT microsatellite PCR kit” (Qiagen) and four primer sets covering the: 16S rRNA (OA2/ Lsc2^12,46^); 16–23s IGS (LpFrag 4-1611F/ LpFrag 4-480R^47^); 50s *rplJ/rplL* (CL514F/ CL514R^48^); and outer membrane protein (OMB1482F/ OMB2086R^5,49^) gene regions. Amplified gene regions were sequenced using “Big-Dye Terminator Cycle Sequencing Kit” (Applied Biosystems) in-house on a “Genetic Analyser 3500xL” (Applied Biosystems). Complementary DNA strands were sequenced separately using forward and reverse primers. Low quality sequence ends were trimmed and contigs were assembled using Clustal-W alignment in “Geneious R11” (Biomatters Ltd.). Lso phylogenies were constructed using the 50s rplJ/rplL gene region to examine the differences between UK Lso haplotypes compared to haplotypes already sequenced globally. 50S gene regions from all available haplotypes and haplotypes found during this study were aligned using Clustal-W. A maximum parsimony tree was constructed on multi-alignments, phylogenetic distance matrices were calculated using Tamura-Nei correction algorithm and clustering was performed using unweighted-pair group method with arithmetic means (UPGMA). A majority rule (50%) consensus tree was produced from 1000 bootstrap replicates. All bioinformatics were performed in “Geneious R11” (Biomatters Ltd.) software.

### Identification of Psyllids

To examine diversity between locations, psyllids were DNA barcoded using one or two gene regions. The intergenic spacer 2 (ITS2) and cytochrome c oxidase subunit 1 (CO1) were amplified and sequenced for identification to species. For amplification of ITS2 primers CAs5p8sFcm-F and CA28sB1d-R^65^ were used and for amplification of CO1 gene regions arthropod barcoding Primers LCO1490 and HCO2198^66^ were used. PCR amplified gene regions were sequenced using the Big-Dye Terminator Cycle Sequencing Kit (Applied Biosystems), forward and reverse complementary DNA strands were sequenced for each sample and analysed using a 3500xL Genetic Analyser (Applied Biosystems). Closest matches were found using BLAST on the GenBank database, BOLD database and our own in-house psyllid barcoding database. Specimens with ≥ 99% similarity were deemed the same species. Specimens that did not match sequences in any of the three databases were identified morphologically.

## Supplementary information

Supplementary file1
