# Supplementary material for: ‘Candidatus Liberibacter solanacearum’ distribution and diversity in Scotland and the characterisation of novel haplotypes from Craspedolepta spp. (Psylloidea: Aphalaridae)
Source: Sci Rep. 2020 Oct 6;10:16567. doi: 10.1038/s41598-020-73382-9 (PMC7538894; doi:10.1038/s41598-020-73382-9)
Supplement: Supplementary file 1 — Supplementary file1 [file 41598_2020_73382_MOESM1_ESM.docx]

**‘*Candidatus* Liberibacter solanacearum’ distribution and diversity in Scotland and the characterisation of novel haplotypes from *Craspedolepta* spp. (Psyllidae: Aphalaridae)**

*Jason C. Sumner-Kalkun^1*^, Fiona Highet^1^, Yvonne M. Arnsdorf^1^, Emma Back^1^, Mairi Carnegie^1^, Siobhán Madden^2^, Silvia Carboni^3^, William Billaud^4^, Zoë Lawrence^1^,* and *David Kenyon^1^.*

**Corresponding author: jason.sumner-kalkun@sasa.gov.scot*

**SUPPLEMENTARY MATERIAL: SUPPLEMENTARY TABLE 1 – NUMBERS OF PSYLLIDS COLLECTED AT EACH LOCATION**

**SUPPLEMENTARY TABLES 2-5 – SINGLE NUCLEOTIDE POLYMORPHISMS IN ‘*CANDIDATUS* LIBERIBACTER SOLANACEARUM’**

|  | Site 1 | Site 2 | Site 3 | Site 4 | Site 5 | Site 6 | Site 7 | Site 8 | Site 9 | Totals |
| --- | --- | --- | --- | --- | --- | --- | --- | --- | --- | --- |
| *Aphalara* sp. | 3 | 5 | 0 | 26 | 4 | 5 | 0 | 7 | 7 | 57 |
| *Arytainilla spartiophila* | 0 | 1 | 1 | 0 | 0 | 0 | 0 | 0 | 1 | 3 |
| *Cacopsylla affinis* | 0 | 4 | 0 | 0 | 0 | 0 | 0 | 0 | 3 | 7 |
| *Cacopsylla ambigua* | 0 | 0 | 2 | 0 | 0 | 0 | 0 | 0 | 0 | 2 |
| *Cacopsylla brunneipennis* | 0 | 0 | 0 | 1 | 0 | 0 | 0 | 0 | 0 | 1 |
| *Cacopsylla mali* | 0 | 0 | 0 | 0 | 0 | 0 | 1 | 0 | 0 | 1 |
| *Cacopsylla melanoneura* | 0 | 15 | 2 | 0 | 0 | 1 | 6 | 0 | 17 | 41 |
| *Cacopsylla peregrina* | 0 | 1 | 0 | 0 | 0 | 0 | 0 | 0 | 6 | 7 |
| *Cacopsylla pulchra* | 0 | 0 | 3 | 3 | 0 | 0 | 0 | 0 | 0 | 6 |
| *Chaemapsylla hartigii* | 0 | 0 | 0 | 0 | 0 | 0 | 0 | 0 | 1 | 1 |
| *Craspedolepta nebulosa* | 0 | 0 | 3 | 0 | 0 | 1 | 0 | 0 | 4 | 8 |
| *Craspedolepta subpunctata* | 0 | 0 | 51 | 0 | 0 | 2 | 2 | 0 | 4 | 59 |
| *Psylla alni* | 8 | 0 | 0 | 0 | 0 | 0 | 0 | 0 | 3 | 11 |
| *Psyllopsis fraxini* | 0 | 0 | 0 | 0 | 0 | 0 | 0 | 0 | 4 | 4 |
| *Psyllopsis fraxinicola* | 0 | 0 | 0 | 0 | 1 | 0 | 0 | 0 | 0 | 1 |
| *Rhinicola aceris* | 0 | 0 | 0 | 0 | 0 | 0 | 0 | 0 | 1 | 1 |
| *Trioza anthrisci* | 0 | 0 | 0 | 50 | 0 | 0 | 0 | 0 | 0 | 50 |
| *Trioza galii* | 0 | 0 | 0 | 0 | 0 | 1 | 0 | 0 | 0 | 1 |
| *Trioza urticae* | 19 | 27 | 1 | 26 | 15 | 48 | 43 | 16 | 79 | 274 |

**Supplementary Table 1. Numbers of psyllids collected at each site.**

| **16S** | **A** | **B** | **C** | **D** | **E** | **F** | **G** | **H** | ***Cras*1a** | ***Cras*1b** | ***Cras*2** | **U** |
| --- | --- | --- | --- | --- | --- | --- | --- | --- | --- | --- | --- | --- |
| 27 | A | A | A | A | G | A | A | A | A | A | A | A |
| 28 | C | C | C | T | C | C | C | C | C | C | C | C |
| 63 | A | A | A | A | G | G | G | G | G | G | G | A |
| 100 | A | A | A | A | A | A | A | G | G | G | G | A |
| 124 | T | G | T | T | T | T | T | T | T | T | T | T |
| 271 | A | A | A | A | A | C | C | A | A | A | A | A |
| 412 | C | C | C | C | C | C | T | C | C | C | C | C |
| 436 | G | G | G | G | G | A | A | G | G | G | G | G |
| 493 | T | C | T | T | T | C | C | C | C | C | C | T |
| 546 | A | A | A | A | A | A | A | A | A | A | A | A |
| 593 | A | A | A | A | A | A | A | A | A | A | A | A |
| 597 | G | G | G | G | G | G | A | G | G | G | G | G |
| 833 | A | A | A | A | A | A | A | G | G | G | G | A |
| 871 | C | C | C | C | T | C | C | C | C | C | C | C |
| 951 | A | A | G | G | A | G | G | A | G | G | G | G |
| 985 | G | G | G | A | G | G | G | G | G | G | G | G |
| 1031 | G | G | G | G/A | G | G | G | G | G | G | G | G |
| 1116 | C | C | C/T | nd | nd | C | nd | T | nd | nd | nd | T |

**Supplementary Table 2.** Single nucleotide polymorphisms in different Lso haplotypes for the 16S rRNA gene. 16S rRNA sequence starting from 1-16 ATCTACCTTTTTCTAC. SNPs are based on the following sequences found in GenBank: A FJ498802 ; B FJ939136 and FJ829813 ; C GU373048 and KX431890 ; D HQ454302 and MG657031; E KF737348 ; F MH259699 ; G MN256492-5; H MK800167; U MG701016.

| **16-23S** | **A** | **B** | **C** | **D** | **G** | **H** | **Cras1a** | **Cras1b** | **Cras2** | **U** |
| --- | --- | --- | --- | --- | --- | --- | --- | --- | --- | --- |
| 39 | A | A | A/G | A | A | A | A | A | A | G |
| 72 | C/T | C | C | C | C | C | C | C | C | C |
| 92 | A | A | A | G | A | A | A | A | A | A |
| 98 | C | C | C | T | C | C | C | C | C | C |
| 177 | A | A | A | A | G | A | A | A | A | A |
| 199 | _ | _ | _ | _ | T | _ | _ | _ | _ | _ |
| 209-210 | _A | GA | GA | _A | GA | AA | _A | _A | AA | _A |
| 219-220 | T_ | TT | T _ | _ _ | _ _ | _ _ | _ _ | _ _ | _ _ | T _ |
| 273 | T | T | C | T | T | T | T | T | T | T |
| 296 | G | A | G | G | G | G | G | G | G | G |
| 368 | T | T | T | T | A | T | T | T | T | T |
| 372 | T | T | T | T | T | C | T | T | T | T |
| 408 | C | T | C | C | C | C | C | C | C | C |
| 434 | G | G | G | A | G | G | G | G | G | G |
| 558 | C | C | C | C | C | T | T | T | T | C |
| 571 | G | A | G | G | G | G | G | G | G | G |
| 592 | T | T | T | T | T | _ | _ | _ | _ | T |
| 613 | C | T | C | C | C | C | C | C | C | C |
| 627 | T | T | T | T | T | T | T | T | T | C |
| 629 | C | C | C | C | C | C | T | T | C | C |
| 647 | A | A | A | A | A/G | A | A | A | A | A |
| 732 | C | C | C | C | A | C | C | C | C | C |
| 806 | C | C | C | C | G | C | C | C | C | C |
| 841 | G | G | G | G | G | G | G | G | G | A |

**Supplementary Table 3.** Single nucleotide polymorphisms in the 16S- 23S rRNA gene of different Lso haplotypes. Nucleotide positions begin at 1-16 TTTATGTTAAGGGCCC. SNPs are based on the following sequences found in GenBank: A GQ381293, GQ926925, and JX624236 ; B FJ830703 ; C JX280523 and MG701020 ; D JX308304 ; G MN256496, MN256497 and MN256498 ; H MK800168; U MG701018. Haplotypes E and F have no associated sequence data available.

| **50S** | **A** | **B** | **C** | **D** | **E** | **F** | **G** | **H** | **Cras1a** | **Cras1b** | **Cras2** | **U** |
| --- | --- | --- | --- | --- | --- | --- | --- | --- | --- | --- | --- | --- |
| 29 | T | T | T | T | T | G | G | T | T | T | T | T |
| 37 | A | A | A | A | A | A | _ | A | A | A | A | A |
| 45 | A | A | A | A | A | A | _ | A | A | A | A | A |
| 54 | G | G | C | G | G | G | G | G | G | G | G | G |
| 90 | G | G | G | G | G | G | G | A | A | A | A | G |
| 93 | A | A | A | G | A | A | A | A | A | A | A | A |
| 111 | C | C | T | C | C | C | C | C | C | C | C | C |
| 135 | G | G | G | G | G | G | G | G | A | G | G | G |
| 140 | G | C | G | G | G | G | G | G | G | G | G | G |
| 145 | A | A | A | A | A | A | A | G | G | G | G | A |
| 150 | A | A | A | A | A | A | A | G | A | A | A | A |
| 160 | C | C | C | T | T | C | C | C | C | C | C | T |
| 162 | G | T | T | G | G | T | T | T | T | T | T | G |
| 166 | G | G | G | G | G | A | A | G | G | G | G | G |
| 168 | A | A | A | A | A | G | G | A | A | A | A | A |
| 171 | A | A | A | G | A | A | A | A | A | A | A | A |
| 179 | A | A | A | A | A | A | A | C | C | C | C | A |
| 183 | G | T | G | G | G | T | T | G | T | G | G | G |
| 193 | G | G | G | G | A | G | G | G | G | G | G | G |
| 220 | C | C | C | A | C | C | C | C | C | C | C | C |
| 251-252 | A_ | A_ | AA | A_/AA | A_ | _ _ | _ _ | A_ | A_ | A _ | A_ | AA |
| 257 | G | A | G | G | G | G | G | G | G | G | G | G |
| 267 | _ _ | _ _ | _ _ | _ _ | _ _ | _ _ | AA | _ _ | _ _ | _ _ | _ _ | _ _ |
| 323 | T | T | T | C | C | T | T | T | T | T | T | T |
| 383 | T | C | C | C | C | C | C | C | C | C | C | C |
| 393-395 | _ _ _ | _ _ _ | GCT | _ _ _ | _ _ _ | GTT | GTT | GCT | _ _ _ | _ _ _ | GCT | _ _ _ |
| 397 | T | C | T | T | T | C | C | C | C | C | C | T |
| 410 | T | T/C | T | T | T | T | T | T | T | T | T | T |
| 415 | C | C | C | C | C | C | C | C | T | C | C | T |
| 432 | G | G | T | G | G | G | G | G | G | G | G | G |
| 438 | G | G | G | G | G | G | G | G | G | G | G | T |
| 464 | T | G | G | G | G | G | G | G | A | A | A | G |
| 470 | A | A | G | A | A | A | A | A | A | A | A | A |
| 482 | T | T | T | T | T | C | T | T | T | T | T | T |
| 497 | T | T | T | T | T | T | T | T | C | C | T | T |
| 512 | A | A | A | A | A | A | A | T | T | T | T | A |
| 518-519 | GA | AA | AA | GA | GA | AG | AG | AG | AG | AG | AG | AA |
| 526 | A | G | A | A | A | A | A | A | A | A | A | A |
| 545 | C | C | C | T | C | C | C | T | T | T | T | C |
| 551 | G | G | G | G | G | G | G | A | A | A | A | G |
| 554 | T | T | T | T | T | T | T | C | C | C | C | T |
| 584 | G | A | G | G | G | A | A | G | G | G | G | G |
| 587-588 | C_ | C_ | C_/CC | C_ | C_ | C_ | C_ | C_ | C_ | C_ | C_ | T_ |
| 600 | G | A | A | A | A | A | A | A | A | A | A | A |
| 615 | A | A | A | A | A | G | G | G | A | A | G | A |
| 621 | G | A | G | G | G | A | A | A | A | A | A | G |
| 630 | A | A | A | A | A | A | A | G | G | G | G | A |

**Supplementary Table 4.** Single nucleotide polymorphisms in 50s rplJ ribosomal gene region of different Lso haplotypes. Nucleotide position in the rplJ/rplL sequence starting from 1-15 GCTAATGATAACAGT (the alanine codon 98 of RplJ). SNPs are based on the following sequences found in GenBank: A EU834131; B FJ498805, FJ498807, JF811598 and JF811599; C LWEB01000001 and GU373051; D HQ454317 and KY777462; E KY777461; F MH259700; G MN256502 and SRR8512499; H MK800169; U MG701023.

| **OMP** | **A** | **B** | **C** | **D** | **F** | **Cras1a** | **Cras1b** | **Cras2** | **U** |
| --- | --- | --- | --- | --- | --- | --- | --- | --- | --- |
| 37 | T | C | T | T | T | T | T | T | T |
| 47 | T | T | T | T | T | G | G | T | T |
| 74 | T | G | T | T | G | G | G | T | T |
| 80 | T | T | T | T | C | T | T | T | T |
| 90 | G | G | G | G | A | G | G | G | G |
| 92 | T | T | T | T | A | T | T | T | T |
| 106 | G | G | G | C | G | G | G | G | G |
| 113 | G | A | G | G | G | G | G | G | G |
| 114 | G | A | A | A | A | A | A | A | A |
| 116 | T | G | T | T | T | T | T | T | T |
| 137 | C | C | C | C | A | C | C | C | C |
| 143 | T | T | T | T | T | T | T | T | G |
| 150 | C | C | C | C | T | C | C | C | C |
| 165 | G | G | A | G | G | G | G | G | G |
| 169 | C | C | T | C | C | C | C | C | C |
| 202 | C | C | A | C | C | C | C | C | C |
| 206 | C | C | T | T | C | C | C | C | T |
| 220 | C | C | T | T | C | C | C | C | T |
| 225 | C | C | C | C | A | C | C | C | C |
| 233 | A | G | G | G | G | G | G | G | G |
| 236 | C | C | C | T | C | T | T | T | C |
| 244 | G | G | A | A | A | A | A | A | A |
| 245 | A | A | C | A | A | A | A | A | A |
| 249 | A | G | A | A | A | A | A | A | A |
| 258 | A | A | A | A | G | A | A | A | A |
| 285 | A | A | A | A | A | G | G | A | A |
| 293 | C | C | C | C | T | C | C | C | C |
| 303 | T | G | T | T | T | T | T | T | T |
| 348 | A | A | A | A | G | A | A | A | A |
| 371 | G | G | G | G | G | A | A | A | G |
| 401 | A | A | A | A | A | G | G | G | A |
| 405 | G | G | T | C | G | G | G | G | T |
| 430 | G | T | G | G | G | G | G | A | G |
| 431 | T | T | G | T | T | T | T | T | T |
| 435 | A | A | A | A | A | G | G | G | A |
| 483 | G | T | T | T | T | T | T | T | T |
| 489 | T | T | T | T | T | A | A | A | T |
| 491 | T | G | T | T | T | T | T | T | T |
| 522 | T | T | T | T | A | T | T | T | T |
| 539 | C | C | C | C | C | T | T | T | C |
| 575 | T | T | C | T | T | T | T | T | T |
| 581 | C | T | C | C | T | C | C | C | C |

**Supplementary Table 5.** Single nucleotide polymorphisms in Outer Membrane Protein gene region for different Lso haplotypes. Nucleotide position begin at 1-14 GGCGTGGTTATAAG. SNPs are based on the following sequences found in GenBank: A JN848754; B KC768327; C KP760078; D KY595979; F MH259701. Haplotypes E, G, and H have not been characterised in the OMP gene region.
